# Draxin regulates hippocampal neurogenesis in the postnatal dentate gyrus by inhibiting DCC-induced apoptosis

**DOI:** 10.1038/s41598-018-19346-6

**Published:** 2018-01-16

**Authors:** Hiroshi Tawarayama, Hirohisa Yamada, Ruhul Amin, Yuiko Morita-Fujimura, Helen M. Cooper, Yohei Shinmyo, Masakado Kawata, Shuntaro Ikawa, Hideaki Tanaka

**Affiliations:** 1 Department of Developmental Neurobiology, Graduate School of Medical Sciences, Kumamoto University, Kumamoto, 860-8556 Japan; 2 Department of Project Programs, Institute of Development, Aging and Cancer (IDAC), Tohoku University, Sendai, 980-8575 Japan; 3 Department of Ecology and Evolutionary Biology, Graduate School of Life Sciences, Tohoku University, Sendai, 980-8578 Japan; 4 Frontier Research Institute for Interdisciplinary Sciences (FRIS), Tohoku University, Sendai, 980-8578 Japan; 5 The University of Queensland, Queensland Brain Institute, Brisbane, Queensland 4072 Australia

## Abstract

Hippocampal neurogenesis in the dentate gyrus (DG) is controlled by diffusible molecules that modulate neurogenic processes, including cell proliferation, differentiation and survival. To elucidate the mechanisms underlying hippocampal neurogenesis, we investigated the function of draxin, originally identified as a neural chemorepellent, in the regulation of neuronal survival in the DG. Draxin was expressed in Tbr2 (+) late progenitors and NeuroD1 (+) neuroblasts in the dentate granule cell lineage, whereas expression of its receptor DCC (deleted in colorectal cancer) was mainly detectable in neuroblasts. Our phenotypic analysis revealed that *draxin* deficiency led to enhanced apoptosis of DCC-expressing neuroblasts in the neurogenic areas. Furthermore, *in vitro* assays using a hippocampal neural stem/progenitor cell (HNSPC) line indicated that draxin inhibited apoptosis in differentiating HNSPCs, which express DCC. Taken together, we postulate that draxin plays a pivotal role in postnatal DG neurogenesis as a dependence receptor ligand for DCC to maintain and promote survival of neuroblasts.

## Introduction

In the hippocampal dentate gyrus (DG), granule cell production begins in the embryo and continues throughout life^1–4^. Accumulating evidence has recently revealed that hippocampal neurogenesis plays a pivotal role in many physiological brain functions, especially those associated with learning and memory. Moreover, altered or impaired neurogenesis is associated with neurological disorders such as Alzheimer’s disease, schizophrenia and depression^5,6^. Intriguingly, a reduction in hippocampal volume and number of newborn dentate granule cells (DGCs) is observed in patients suffering these diseases, as well as in the relevant animal models. Conversely, enhanced neurogenesis in the hippocampus is seen in other neurological diseases such as the epilepsy, ischemia and traumatic injury^7,8^. These opposing observations imply that the disruption of different molecular pathways is likely to regulate hippocampal neurogenesis in each neuropathological condition. To date, elucidation of the mechanisms underlying hippocampal neurogenesis has identified a variety of diffusible factors able to modulate key hippocampal neurogenic processes, including cell proliferation, differentiation and survival^9,10^. In this study, we further expand our understanding of hippocampal neurogenesis through the identification of the axon guidance cue, Draxin, as an important regulator of neuronal precursor survival.

The neural chemorepellent draxin, which we previously isolated using a signal sequence trap, is indispensable for proper navigation of growing axons and migrating neurons in developing embryos^11–20^. Furthermore, our study revealed that draxin is important not only for axon navigation but also for hippocampal development. Draxin loss leads to enhanced apoptosis at embryonic day 18 (E18), impaired DG development, fewer dentate granule cells and reduced DG size in juveniles^20^. However, little is known regarding the underlying mechanism responsible for such DG phenotypes in *draxin* knockout (KO) mice. Previous studies reported that draxin interacts with netrin receptors physically, although only DCC (deleted in colorectal cancer) and neogenin were proven to mediate the inhibitory effect of draxin^18,21^. These transmembrane molecules are also known as dependence receptors, which trigger neuronal apoptosis and promote survival in the absence and presence of their ligands, respectively^22–27^. Thus, one possible explanation for the DG phenotype in *draxin* KO mice described above is deregulation of these dependence receptors due to lack of draxin. In the present study, we elucidated the cellular and molecular mechanisms underlying draxin-regulated hippocampal neurogenesis by investigating the role of draxin in dependence receptor-induced apoptosis.

## Results

### Expression of draxin and its receptors in the postnatal dentate gyrus

**Figure 1:**
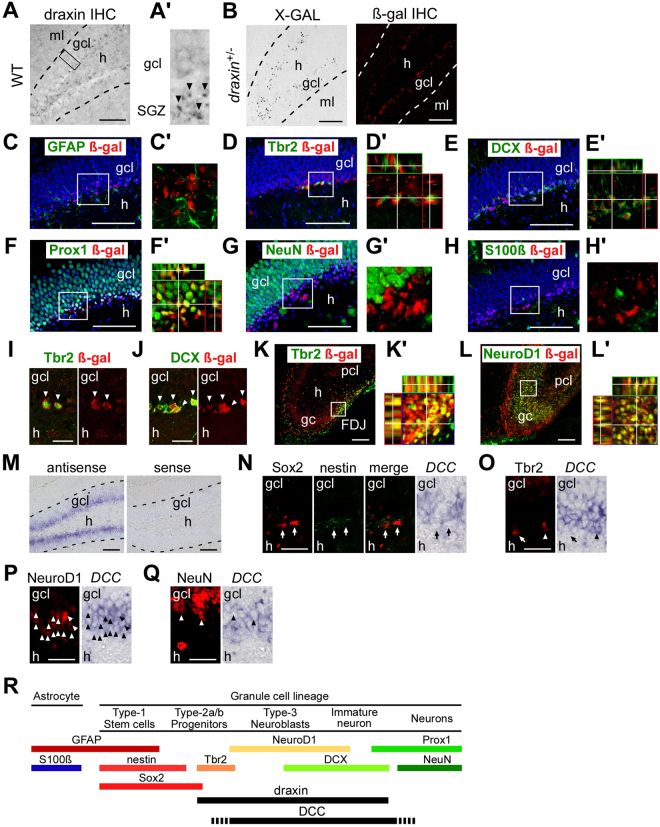
Expression of draxin and its receptor DCC (deleted in colorectal cancer) in the postnatal sub-granular zone. (**A**) Immunohistochemical localization of endogenous draxin at postnatal day (P) 30. A’, Higher magnification of the boxed region in A. Arrowheads indicate draxin immunoreactivity. (**B**) Cells expressing β-gal detected by X-GAL staining (left) and immunohistochemistry (right) on the dentate gyrus (DG) of P30 mutant mice heterozygous for *draxin* (*draxin*^+/−^). The exon including the translation start site of the *draxin* gene was replaced with a β-gal expression cassette in either allele. (**C–L**) Confocal images of the *draxin*^+/−^ DG double-labeled for β-gal and either granule cell lineage markers or glial cells, including GFAP (**C**,C’), Tbr2 (**D**,D’,**I**,I’,**K**,K’), NeuroD1 (**L**,L’), DCX (**E**,E’,J,J’), Prox1 (**F**,F’), NeuN (**G**,G’) and S100β (**H**,H**’**), at P30 (**C–H**), 3 month-old (**I**,**J**), and P2 (**K**,**L**). The boxed areas in (**C–H**,**K**,**L**) are magnified in (C’-H’,K’,L**’**), respectively. In (**I** and **J)**, merged images of the red (β-gal) and green (marker) channels, and the red channel (β-gal) alone, are provided in the left and right panels, respectively. Arrowheads in (**I** and **J**) indicate colabeled cells. Some sections were counterstained with DAPI. The β-gal-immunoreactive cells are considered draxin-expressing, and are mainly restricted to Tbr2-expressing late progenitors and DCX/NeuroD1-expressing neuroblasts, regardless of developmental stages. (**M**) *In situ* hybridization analysis of DCC on the P30 DG using antisense or sense probes. (**N–Q**) Double staining of *in situ* hybridization for *DCC* and immunochemistry for granule cell lineage markers including GFAP and nestin (N), Tbr2 (**O**), NeuroD1 (**P**) and NeuN (**Q**) at P30. Arrowheads and arrow in (**N–Q**) indicate cells expressing both of DCC and markers, and marker only, respectively. (**R**) Schematic showing expression of the marker molecules, draxin and DCC in astrocytes and cells in the granule cell lineage during differentiation. gcl: granule cell layer; h: hilus; ml: molecular layer; FDJ: the fimbriodentate junction; gc: granule cell; pcl: pyramidal cell layer. These abbreviations also apply to the other figures in this paper. Scale bars: (**A**–**J**,**M**) 100 µm; (**K**,**L**,**N–Q**), 20 µm.

Since impairment of DG development in *draxin* KO mice is obvious at early postnatal stages and thereafter, but not E17.5 (Supplementary Figure S1), we first analyzed the expression of draxin and its candidate receptors in the DG at the postnatal stages to delineate the mechanism underlying the draxin-mediated regulation of DG development. Given our observation that draxin expression was restricted to the subgranular zone (SGZ; the innermost part of the granule cell layer) of the DG in juveniles (Fig. 1A,A’,B), we sought to determine the type of cells expressing draxin in the SGZ. To do this, hippocampal sections from postnatal day 30 (P30) mutant mice, heterozygous for *draxin*, were immunostained with antibodies to β-gal and various molecular markers for the granule cell lineage or glial cells. The *draxin* mutant mice used in this study were generated by replacing the second exon of the *draxin* gene containing the translation start site with a β-gal expression cassette^11^. Thus, expression of β-gal can be considered to mimic that of endogenous draxin. On the other hand, cells classified as in the granule cell lineage can be further categorized into several groups according to their expression of marker genes: neural stem cells (type-1), progenitors (type-2a/b), neuroblasts (type-3), immature and mature granule cells^28^ (also see Fig. 1R). In this study, we regarded cells expressing GFAP as neural stem cells, nestin (+) or Sox2 (+) cells as neural stem/early progenitor cells, Tbr2 (+) cells as late progenitors, NeuroD1 (+) or doublecortin (DCX) (+) cells as neuroblasts, and NeuN (+) cells as mature neurons. The β-gal immunoreactive (i.e., draxin-expressing) cells were mainly restricted to Tbr2-immunoreactive late progenitors (type-2b), and DCX-immunoreactive neuroblasts (type-3) and immature neurons (Fig. 1D,D’,E,E’). β-gal expression was also observed in a small fraction of the cells expressing the pan-dentate granule cell marker Prox1, representing mainly post-mitotic neurons (Fig. 1F,F’). In contrast, β-gal expression was hardly detectable in neural stem cells and mature granule cells immunoreactive for GFAP and NeuN, respectively (Fig. 1C,C’,G,G’). Also note that β-gal (+) cells were not present in glial cells and mature astrocytes, as determined by GFAP and S100β immunoreactivity, respectively (Fig. 1C,C’,H,H’). A similar pattern of draxin expression was observed at other developmental stages. Almost all the Tbr2 (+) late progenitors, and NeuroD1 (+) neuroblasts co-expressed draxin in the SGZ of the 3-month-old adult DG (Fig. 1I,J). In the DG of P2 pups, draxin was expressed in the Tbr2 (+) cells located in the fimbriodentate junction (FDJ; Fig. 1K,K’), which is a transient neurogenic zone containing abundant undifferentiated neuronal cells^29^. Also in the P2 pups, draxin was expressed in neuroblasts expressing NeuroD1 (Fig. 1L,L’). The specific expression of draxin in late progenitors and neuroblasts suggests that draxin may play a similar role in hippocampal neurogenesis regardless of developmental stage.

We next investigated the expression of DCC and its closely-related molecule neogenin, which were previously shown to mediate draxin-induced repulsion and attraction^18,21^ in the SGZ of P30 juvenile mice. *DCC* mRNA was localized in the inner part of the dentate granule cell layer including the SGZ (Fig. 1M). Furthermore, double staining with the antibodies to the markers for the granule cell lineage confirmed that *DCC* is expressed in almost all NeuroD1 (+) neuroblasts (Fig. 1P), and a small population of Tbr2 (+) late progenitors (Fig. 1O) and NeuN (+) mature neurons (perhaps young mature neurons; Fig. 1Q), but not early progenitors immunoreactive to Sox2 and nestin (Fig. 1N). Compared with *DCC*, *neogenin* was expressed more widely in the granule cell lineage including early/late progenitors, neuroblasts and immature neurons (Supplementary Figure S2).

### Enhanced apoptosis in *draxin* knockout mice

**Figure 2:**
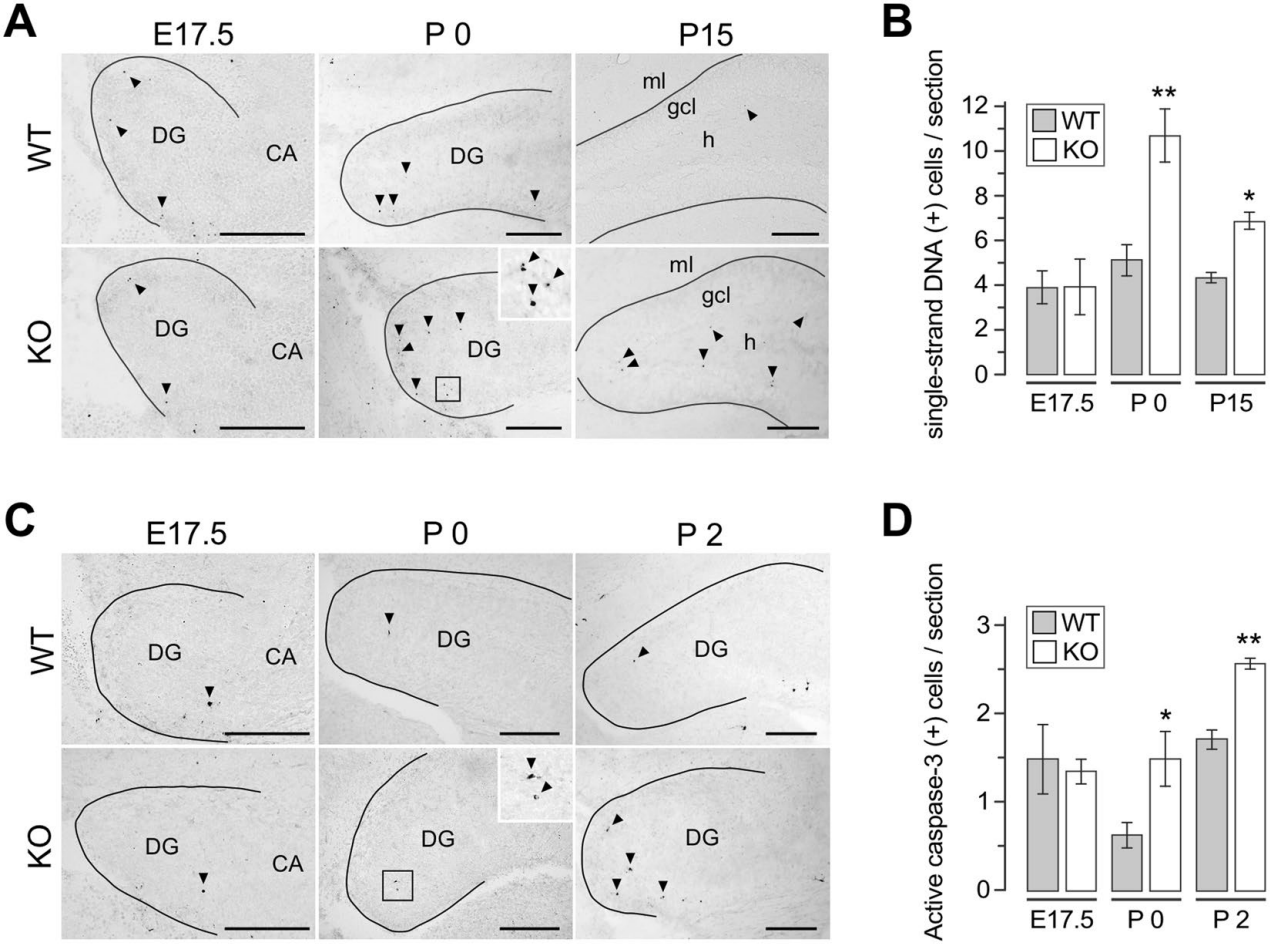
Increased apoptosis in *draxin*-deficient mice. (**A**) Apoptosis in the draxin wild-type (WT) and knockout (KO) DG detected by immunohistochemistry for single-strand DNA at E17.5, P0 and P15. (**B**) Quantification of ssDNA-immunoreactive cells in the entire DG at E17.5 and P0, and in the subgranular zone (SGZ) at P15. (**C**) Immunostaining of active caspase-3 on the WT and KO DG at E17.5, P0 and P2. (**D**) Quantification of AC-3-immunoreactive cells in (**C**). Black lines in (**A** and **C**) emphasize the pial surface of the DG. The boxed areas in (**A** and **C**) are magnified in the upper right of panels. Arrowheads indicate immunoreactive cells. The average number of immunoreactive cells was calculated from 5 independent brains per group. Error bars indicate s.e.m. *p < 0.05, **p < 0.01 against WT mice (Student’s t-test). CA: Cornu Ammonis. Scale bars, 200 µm.

**Figure 3:**
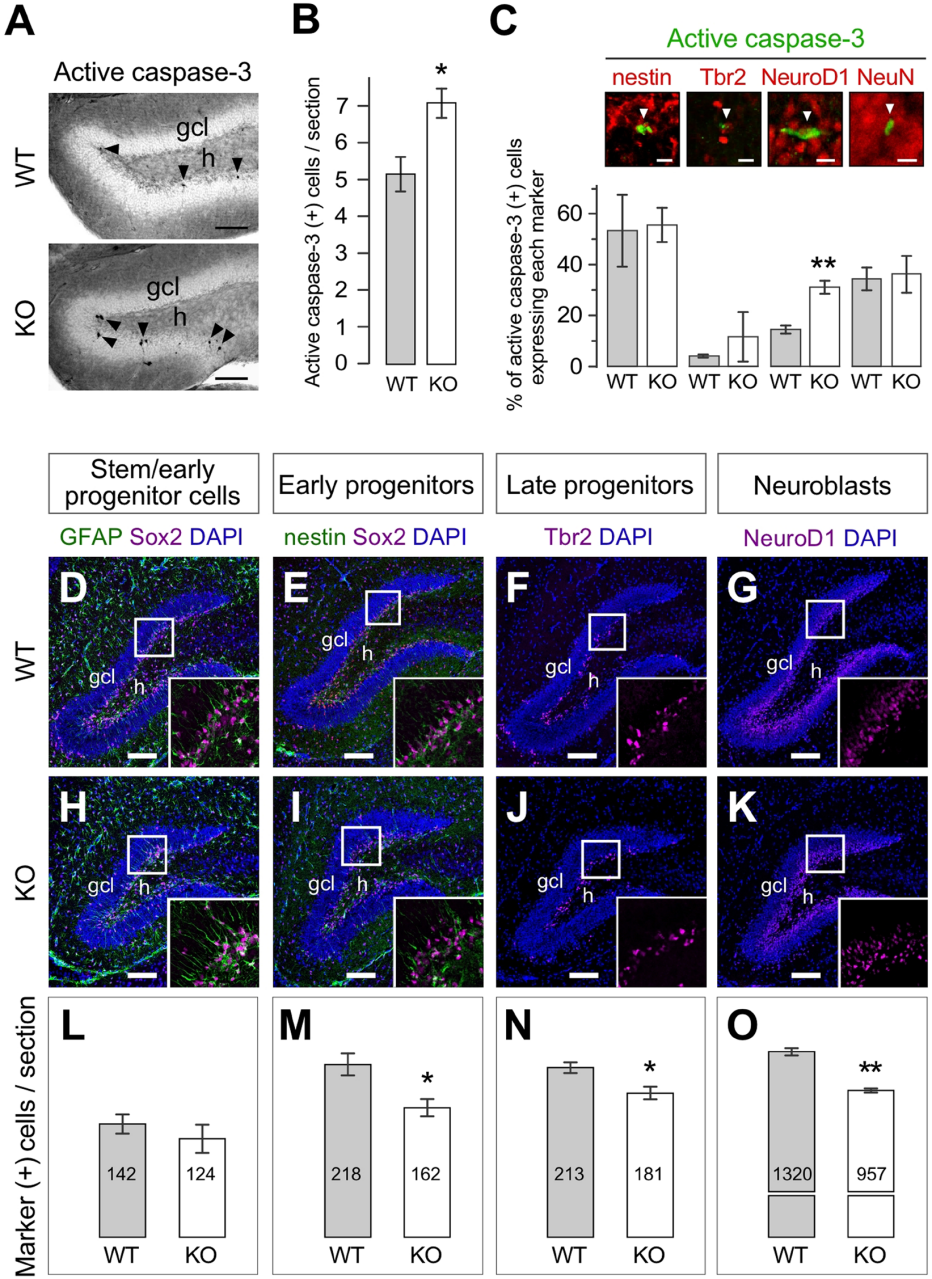
Excessive apoptotic neuroblasts and reduction of cells in the granule cell lineage in the *draxin* KO SGZ. (**A**) Immunostaining of the P21 DG for active caspase-3. Arrowheads indicate apoptotic cells. (**B**) Quantification of immunoreactive cells in (**A**) Apoptotic cells were remarkably increased in KO mice (n = 6). (**C**) Confocal images of SGZ cells double-labeled for active caspase-3 and granule cell lineage markers. Over 150 active caspase-3-immunoreactive cells per mouse were counted for each marker (n = 4). Note that NeuroD1-expressing neuroblasts, but not nestin-expressing neural stem/progenitor cells or NeuN-expressing mature neurons, underwent excessive apoptosis in the KO SGZ. Number of apoptotic cells tends to increase in late progenitors expressing Tbr2 in *draxin* KO mice, but it was not significant from that in WT mice. (**D–K**) Confocal images of DG sections of P21 WT (**D**–**G**) and KO (**H–K**) mice immunostained with markers for neural stem cells (GFAP and Sox2; **D** and **H**), neural stem/early progenitor cells (nestin and Sox2; **E** and **I**), late progenitor cells (Tbr2; **F** and **J**), and neuroblasts (NeuroD1; **G** and **K**). Immunoreactive cells were quantified in **L*–*O**, respectively. *Draxin* deficiency drastically decreased the number of progenitors and neuroblasts (n = 4). Error bars indicate s.e.m. *p < 0.03, **p < 0.01 (Student’s t-test). Scale bars: 100 µm.

Although we previously reported that *draxin* deficiency results in increased apoptosis in the hippocampal DG at an embryonic stage E18.5^20^, the apoptotic phenotype at earlier embryonic and postnatal stages has not been analyzed. Therefore, we monitored apoptosis again from embryonic to early postnatal stages in *draxin* KO mice. Apoptotic cells were identified by immunostaining with the single-stranded DNA antibody, which can recognize single-stranded DNA fragments consisting of various base sequences and has previously shown to be available for detection of apoptotic cells as well as the TUNEL assay^30–33^. Excessive apoptosis was detected in DG cells of *draxin* KO mice at P0 and P15, but not E17.5 (Fig. 2A,B). Interestingly, apoptotic cells were observed in the neurogenic areas, characterized by abundant multi- and uni-potent neural cells, including in the subpial zone and hilus of the DG at E17.5 and P0^29^, and the SGZ at P15. Furthermore, the immunohistochemical analysis using another apoptotic marker active (cleaved) caspase-3 confirmed that apoptosis was not observed in *draxin* KO mice at E17.5 but is present at early postnatal stages P0, P2 and P21 (Fig. 2C,D; also see Fig. 3A,B).

### Excessive apoptosis of neuroblasts in the subgranular zone of *draxin* knockout mice

Given that draxin loss leads to enhanced apoptosis in DG cells, we next identified the specific apoptotic cell type. To this end, we co-immunostained hippocampal slices with granule cell lineage markers and active caspase-3. Active caspase-3 (+) cells are still detectable in the SGZ of *draxin* KO mice even at a juvenile stage P21 (Fig. 3A,B). Furthermore, co-staining with granule cell lineage markers revealed that the number of apoptotic neuroblasts immunoreactive for NeuroD1 was 2.5-fold greater in the KO compared to wild-type (WT) mice (Fig. 3C). In contrast, no significant difference was observed in neural stem/early progenitors, late progenitors, or mature granule cells (n = 4; at least 150 cells immunoreactive for active caspase-3 were analyzed for each marker per each genotype; Fig. 3C).

We next counted the number of cells reactive to antibodies that recognize the various marker molecules for granule cell differentiation in the P21 DG of *draxin* KO mice, to investigate the impact of *draxin* deficiency on the granule cell population. As expected, the number of NeuroD1(+) neuroblasts exhibiting excessive apoptosis was significantly decreased in the KO SGZ (Fig. 3G,K,O). Neural stem/early progenitors expressing nestin and Sox2 (Fig. 3E,I,M), and late progenitors expressing Tbr2 (Fig. 3F,J,N), were also reduced in number in the KO DG, even though no differences in the apoptotic indices were observed in nestin- and Tbr2-expressing cells (Fig. 3C). On the other hand, no significant differences between KO and WT mice were observed in the number of neural stem cells doubly immunoreactive for GFAP and Sox2 (Fig. 3D,H,L).

### Draxin suppresses DCC-induced apoptosis

**Figure 4:**
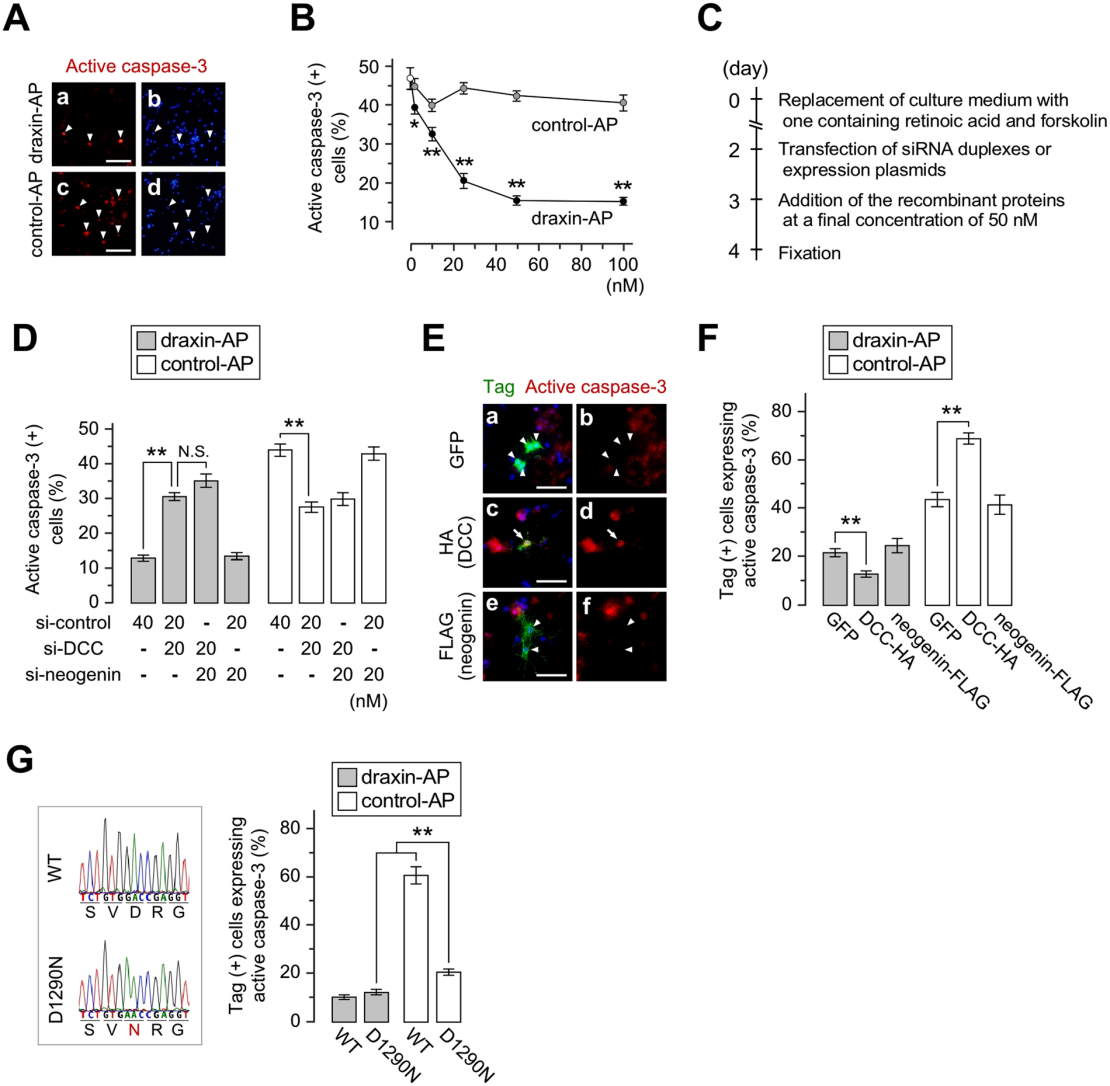
Effects of draxin and its receptors on apoptosis of differentiating hippocampal neural cells. (**A**) Active caspase-3 immunostaining on differentiating rat hippocampal neural stem/progenitor cells (HNSPCs) cultured in medium containing alkaline phosphatase (AP)-fused draxin (draxin-AP; **a** and **b**) or AP alone (control-AP; *c* and *d*) at the concentration of 50 nM. Immunoreactivity to active caspase-3 (red) and DAPI staining (blue) in the same microscopic field are shown in *a*/*b*, and *c*/*d*. (**B**) Quantification of apoptotic cells treated with various concentrations of draxin-AP or control-AP. (**C**) Schematic of the experimental time course for gain- and loss-of-function studies. (**D**) Effects of DCC and/or neogenin knockdowns on apoptosis in the presence or absence of draxin. Differentiating HNSPCs transfected with si-DCC and/or si-neogenin were cultured in medium containing 50 nM of draxin-AP or control-AP, and then analyzed for apoptosis. (**E**) Costaining of cells transfected with GFP (*a* and *b*), HA-tagged DCC (**c** and **d**), and FLAG-tagged neogenin (*e* and *f*) plasmids with active caspase-3, in the absence of exogenous draxin. Merged images of red (active caspase-3) and green (tags) channels are shown in *a*, *c*, *e*, and the red channel alone in *b*, *d*, *f*. Arrowheads and arrows indicate tag-expressing living cells and tag/active caspase-3-expressing dying cells, respectively. (**F**) Overexpression effects of DCC or neogenin on apoptosis. The tagged transfectants above were cultured in medium containing draxin-AP or control-AP (50 nM), and then active caspase-3 (+) cells expressing tags were quantified. (**G**) Implication of caspases in DCC-induced apoptosis. A target site of caspases in the cytoplasmic domain of DCC, aspartic acid (D) at amino acid 1290, was substituted for asparagine (N; left panel), and the mutated (D1290N) or WT DCC was overexpressed in HNSPCs in the presence of draxin-AP or control-AP (50 nM) to investigate relevance of caspases to DCC-induced apoptosis (right panel). The average number of immunoreactive cells was calculated from 5 independent experiments in (**B**,**D**,**F**,**G**). Error bars indicate s.e.m. *p < 0.03, **p < 0.01 (Student’s t-test in (**B**,**G**); Tukey-Kramer test in (**D** and **F**). N.S.: not significant. Scale bars: (**A**) 100 µm; (**E**) 50 µm.

We next investigated the effect of draxin on apoptosis using hippocampal neural stem and progenitor cells (HNSPCs) derived from the juvenile/adult rat (see M&M). When the rat-derived HNSPCs were cultured in medium containing the differentiation inducers, retinoic acid (RA) and forskolin (FK), for 4 days, expression of nestin was rapidly downregulated, while that of an early neuronal marker Tuj1 was gradually upregulated in almost all the cells (Supplementary Figure S3A). Additionally, expression of the draxin receptors, DCC and neogenin, was upregulated drastically on the first day of culturing in medium containing the differentiation inducers and thereafter (Supplementary Figure S3B). After culturing in medium containing RA/FK/B-27 supplements for 2 days, differentiating HNSPCs were treated with various concentrations of recombinant alkaline phosphatase (AP)-fused draxin (draxin-AP), or AP alone as a negative control (control-AP) in medium without RA/FK/B-27 supplements for an additional day. In the absence of draxin, 46.8% of the cells underwent apoptosis, as determined by immunoreactivity to active caspase-3 (Fig. 4A,B), whereas the addition of draxin-AP rescued the apoptotic phenotype in a dose-dependent manner (Fig. 4A,B).

We next conducted loss-of-function experiments for DCC and neogenin using the siRNA transfection system, in which siRNA duplexes were introduced into 77.4% of differentiating HNSPCs (Supplementary Figure S4A,B), to investigate their roles in draxin-mediated apoptotic regulation. Downregulated expression of target genes in siRNA transfectants was confirmed at the protein level as shown in Supplementary Figure S4C and D. Either draxin-AP or control-AP (50 nM) was added to the medium 24 h after si-DCC and/or si-neogenin were transfected into differentiating HNSPCs, and then the transfectants were cultured for an additional day (Fig. 4C). Active caspase-3 staining revealed that knockdown of DCC but not neogenin resulted in an attenuation of draxin-induced neuronal survival (Fig. 4D, *grey bars*). Furthermore, knockdown of neogenin in addition to DCC did not lead to further attenuation of draxin-induced neuronal survival compared to the single-knockdown of DCC when draxin was present (Fig. 4D, *grey bars*). On the other hand, DCC knockdown in the absence of draxin resulted in the opposite effect on neuronal survival. DCC knockdown significantly increased the viability of differentiating HNSPCs (Fig. 4D, *white bars*). In contrast to DCC, knockdown of neogenin never affected cell survival (Fig. 4D).

We next examined the effects of overexpression of DCC and neogenin on neuronal survival. The differentiating HNSPCs pretreated with RA/FK for one day were transfected with each of the expression plasmids encoding HA-tagged DCC, FLAG-tagged neogenin, or GFP as a negative control, and then treated with draxin-AP or control-AP for 24 hours after transfection. Overexpression of DCC resulted in the opposing effects, depending on the presence of draxin. DCC induced apoptosis in the absence of draxin (Fig. 4E,F, *white bars*), but suppressed it in the presence of the ligand (Fig. 4F, *gray bars*). In contrast, neogenin overexpression had no effect on the viability in the presence or absence of draxin (Fig. 4F). We next investigated the relevance of caspases to DCC-induced apoptosis in the absence of draxin by substituting the amino acid at position 1290 from aspartic acid to asparagine, which is a cleavage target for caspases. Overexpression of a mutant form of DCC (D1290N) failed to trigger apoptosis in differentiating HNSPCs without draxin, although it still retained the prosurvival activity in the presence of draxin (Fig. 4G).

These data indicate that draxin functions as a prosurvival ligand for DCC, which functions as a dependence receptor to induce apoptosis in the absence of draxin. Our data also suggest that DCC-induced apoptosis requires caspase-mediated cleavage of the intracellular domain in DCC.

## Discussion

This study is the first to provide evidence demonstrating draxin-mediated inhibition of DCC-induced apoptosis in neuronal precursors of the DG, which ensures expansion of dentate granule cell population in the developing hippocampus. Previous studies demonstrated the relevance of caspases to DCC-induced apoptosis using the immortalized cell line, HEK 293 T^25,34^. In accordance with this observation, our findings showed that a mutation in the caspases cleavage site (amino acid 1290) in DCC resulted in decreased apoptosis in differentiating HNSPCs *in vitro*, suggesting that DCC-induced apoptosis in the absence of ligands is also mediated through a caspase-dependent mechanism. It has been proposed that inhibition of apoptosis is dependent on conformational change which masks the caspase cleavage site on DCC upon Netrin-1 binding^25^. Therefore, binding of draxin to DCC might also result in a conformational change, leading to apoptotic inhibition due to the inaccessibility of the caspase target in the DCC intracellular region. Based on the *in vitro* data obtained in the present study, we believe that the draxin-mediated attenuation of the DCC-induced HNSPC death is mainly due to inhibition of a caspase-dependent apoptosis in the presence of its ligand. This hypothesis is supported by our observation that cell death was drastically prevented in HNSPCs expressing a caspase-resistant DCC mutant compared to the wild-type in the absence of draxin (white bars in Fig. 4G; about 20% and 60% of cell death for a mutant and the wild-type DCC, respectively, i.e., 40% of cell death inhibition). Thus, we have elucidated the principle mechanism underlying the draxin-mediated inhibition of the DCC-induced HNSPC death. In the present study, we also found that knockdown of DCC resulted in increased apoptosis in HNSPCs even in the presence of draxin. It is previously reported that dependence receptors, including DCC, not only trigger apoptosis in the absence of their ligands, but can also transduce cell survival-promoting signals in response to their ligands, depending on the biological context^22,26,27,35,36^. Therefore, as suggested by our data, it is possible that DCC positively regulates survival of HNSPCs through interaction with draxin and/or the other ligands such as netrin.

We previously revealed molecular interactions between draxin-AP and netrin receptors, such as DCC and neogenin, using saturation binding curves and Scatchard analyses^18,21^. In contrast to our findings, other research groups have reported that draxin does not interact with netrin receptors, but instead, with Netrin-1 itself^37,38^, using different experimental approaches from ours. If correct, draxin would be expected to attenuate Netrin-1 signals by preventing Netrin-1 from interacting with the netrin receptors, and consequently inhibit the prosurvival effect of Netrin-1 on DCC-expressing cells. However, the draxin-mediated attenuation of the prosurvival activity of Netrin-1 in this context cannot explain the inhibition of DCC-induced apoptosis in the presence of exogenous draxin, as demonstrated in the present *in vitro* study. Furthermore, Netrin-1-mediated prosurvival activity would be predicted to become predominant in the absence of draxin, resulting in fewer apoptotic cells in the *draxin* KO SGZ. However, robust apoptosis was observed in *draxin* KO mice. Taken together, we believe that draxin regulates the proapoptotic activity of DCC in the SGZ by directly interacting with the DCC dependence receptor and not by preventing the interaction between prosurvival Netrin-1 and DCC.

Immunohistochemical and *in situ* hybridization analyses performed in this study indicated that both draxin and DCC are expressed in NeuroD1 (+) or DCX (+) neuroblasts, and that draxin and decreased levels of DCC are expressed in Tbr2 (+) late progenitors. Furthermore, expression of draxin and DCC is below the detection limit in neural stem cells/early progenitors and mature granule cells. Since robust apoptosis was observed only in neuroblasts in *draxin* KO mice, DCC expression would explain why neuroblasts but not the cells representative of other differentiation stages in the granule cell lineage undergo apoptosis in the SGZ of *draxin* KO mice. Based on these findings, we propose that draxin secreted from late progenitors and neuroblasts inhibits apoptosis of DCC-expressing neuroblasts in the DG via autocrine and possibly paracrine signaling.

**Figure 5:**
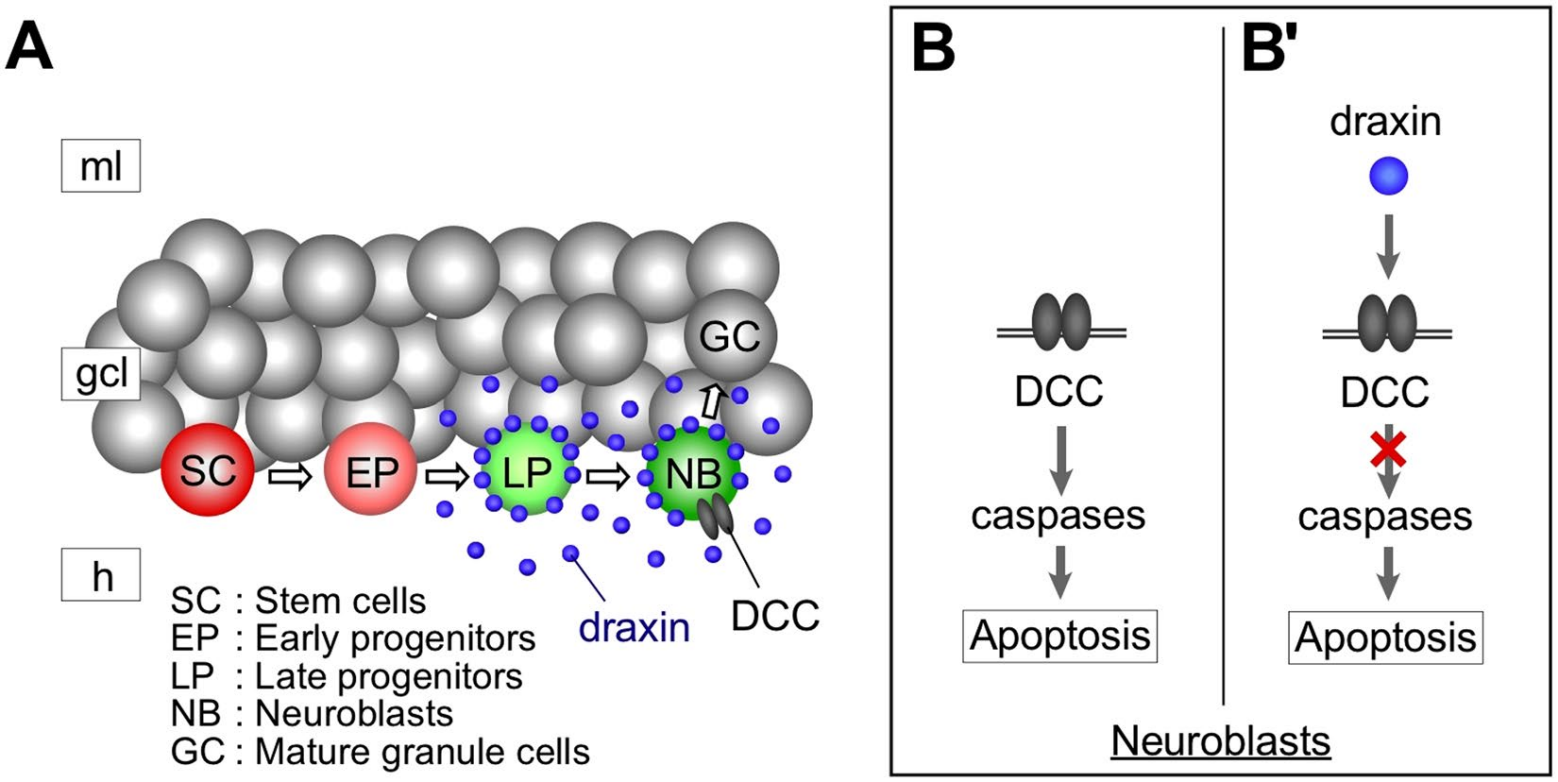
A model for draxin function in postnatal neurogenesis of the DG. In the neurogenic regions of the postnatal DG, draxin is expressed only in Tbr2 (+) late progenitors and DCX/NeuroD1 (+) neuroblasts, but not in other granule cell lines or glial cells (**A**). On the other hand, the draxin receptor DCC, a dependence receptor, is principally expressed in neuroblasts (**A**), where it induces apoptosis in a caspase-dependent manner in the absence of draxin (**B**). However, the DCC-induced apoptosis is prevented in the presence of draxin (B’). Thus, draxin secreted from late progenitors and neuroblasts could prevent neuroblasts from undergoing DCC-induced apoptosis during differentiation.

The present and previous studies revealed that draxin is necessary for DG morphogenesis from early postnatal to adult stages throughout life^20^. Excessive apoptosis of cells in the granule cell lineage would generally result in a reduced number of mature dentate granule cells that represent a considerable portion of the DG, leading to DG malformation. This is mechanistically supported by studies of mice lacking genes such as Sox2, Tbr2, Prox1 and neurogenin2, which are known to be essential for hippocampal neurogenesis. In each case, the neuronal population was drastically decreased due to excessive apoptosis, leading to reduced size of the DG^39–42^. The malformation of the DG in *draxin* KO mice may also be attributable in part, to the deregulation of neurogenic processes including neuronal proliferation and differentiation. Previous studies indicated that canonical Wnts play pivotal roles in regulating neuronal proliferation and differentiation in the developing DG^43–46^, and that Neucrin, a zebrafish homologue of draxin, attenuates the canonical Wnt/β-catenin signaling by competing with Wnts for their receptor component, lipoprotein receptor-related protein 6 (LRP6)^47,48^. Thus, deletion of the *draxin* gene may lead to defective neuronal proliferation and/or differentiation of SGZ cells due to deregulation of the canonical Wnt signals. Supporting this, the present study indicated that *draxin* deficiency resulted in reduction of not only neuroblasts, but also early/late progenitors, which do not express DCC or exhibit excessive apoptosis. Future studies would focus on the impact of loss of the *draxin* gene on Wnt-driven proliferation/differentiation of SGZ cells to further elucidate the functions of draxin in hippocampal neurogenesis. In summary, we have identified the cellular and molecular mechanisms underlying draxin-mediated regulation of neuronal survival in the postnatal SGZ, and found that draxin acts as a dependence ligand for DCC to prevent the proapoptotic effect of DCC in neuroblasts (Fig. 5). This suggests that draxin is essential for expansion of the neuronal population in the developing DG.

## Materials and Methods

### Animals

*Draxin* mutant mice were previously described^11^. All animal experiments and animal care were performed according to the procedures and guidelines approved by the Committees on Animal Research at Kumamoto and Tohoku Universities.

### Immunochemical analysis

Immunostaining of tissue sections and cultured cells were performed following the procedures described in the Supplementary Text.

### *In situ* hybridization

*In situ* hybridization (ISH) was performed using the digoxygenin (DIG)-labeled cRNA probes corresponding to 3384–3982 bases of the mouse *DCC* (NCBI Reference Sequence: NM_007831.3) on hippocampal slices (200 µm) obtained from juvenile mice, respectively, following the procedure reported before^49^. To perform double staining with immunohistochemistry, hippocampal slices were cut into 10 µm sections using a cryostat (Leica) after developing color for ISH, and then sections were immunostained following the procedure described in the Supplementary Text.

### Cell culture

Hippocampal neural stem and progenitor cells (HNSPCs) derived from the juvenile/adult rat (P43-55; Merck-Millipore, SCR022) were cultured in a mixture of Dulbecco’s Modified Eagle’s Medium and Ham’s Nutrient Mixture F-12 (DMEM/F-12; Wako Pure Chemical) containing B-27 supplement (Invitrogen), human basic fibroblast growth factor (FGF-2; 20 ng/ml; PeproTech) and human epidermal growth factor (EGF; 20 ng/ml; PeproTech), in a CO_2_ incubator at 37 °C. Cells were sub-cultured upon reaching 80% confluency using Accutase (Innovative Cell Technologies).

### Plasmid construction and site-directed mutagenesis

The neogenin expression plasmid was constructed by ligating the FLAG tagged-DNA fragment containing the entire mouse neogenin (NM_008684: 314 - 4789) coding region amplified from cDNA prepared from P0 whole mouse brains using KOD DNA polymerase (Toyobo) into Hind III and Not I sites of the pMX-CAG vector (Cell Biolabs). The sequence of the constructed plasmids was confirmed by sequencing. The rat DCC expression plasmid was constructed by inserting the Sal I-Not I HA-tagged rat DCC fragment derived from pcDNA-rat DCC-HA (kindly gifted from Dr. Alain Chédotal) into Xho I and Not I sites of the pMX-CAG vector (Cell Biolabs; pMX-CAG-rat DCC-HA). To substitute aspartic acid for asparagine at the 1290^th^ amino acid residue of DCC, a PCR-based site-direct mutagenesis was performed on pMX-CAG-rat DCC-HA using KOD Plus Mutagenesis Kit (Toyobo) according to the instruction. The sequence of the primers used for the mutagenesis was shown below. 5′-AACCGAGGTTTCGGAGCAGGAAGAA-3′ and 5′-CACAGACAGTGTTGGGAATGGTACTGG-3′.

### Transfection of siRNA duplexes or expression plasmids

Knock-down of gene expression was achieved by introduction of siRNA duplexes (Sigma-Aldrich) designed for the draxin receptors, DCC and neogenin, into rat HNSPCs using the Lipofectamine RNAiMax Reagent (Invitrogen) according to the instructions. The sequences of siRNA duplexes used in this study were: 5′-CAGUGAACGGCUCCCAUAATT-3′ and 5′-UUAUGGGAGCCGUUCACUGTT-3′ for rat neogenin; and 5′-CUAUGUAUUACUUUCGAAUTT-3′ and 5′-AUUCGAAAGUAAUACAUAGTT-3′ for rat DCC. Overexpression was achieved by introduction of the expression plasmids described above into rat HNSPCs using the NanoJuice Transfection Kit (Merck-Millipore) according to the instruction.

### Cell death assay

Rat HNSPCs were cultured in DMEM/F-12/B-27 medium containing the differentiation-inducing compounds, retinoic acid (RA: 1 µM; Sigma-Aldrich) and forskolin (FK: 5 µM; Wako Pure Chemical), to direct neuronal differentiation. On the 3^rd^ day of culturing, medium was replaced with DMEM/F-12 medium containing various concentrations of the recombinant proteins for human alkaline phosphatase (AP) and AP-fused draxin (draxin-AP) without B-27 supplement and differentiation inducers, and then cells were cultured for one more day. After fixation with 4% PFA in PBS, the recombinant protein-treated cells were immunostained with antibodies to an apoptosis marker, active caspase-3, following the procedure described in the Supplementary Text. Production of the AP and draxin-AP recombinant proteins were described before^11^. In some experiments, cells were transfected with siRNA duplexes or expression plasmids on the 2^nd^ day of culturing with RA/FK-containing medium, and then cultured for one more day in the presence of 50 nM of the recombinant proteins for draxin-AP and AP, to determine whether DCC and neogenin are implicated in transducing draxin signals.

### Statistics

Quantitative data were analyzed using Student’s t-test for a paired sample group or Tukey-Kramer test following ANOVA for more than three sample groups, with JMP Pro 12 software (SAS Institute Inc.).

## Electronic supplementary material

Supplementary information

## References

[1] Bayer SA (1982). Changes in the total number of dentate granule cells in juvenile and adult rats: a correlated volumetric and 3H-thymidine autoradiographic study. Exp Brain Res.

[2] Kaplan MS, Hinds JW (1977). Neurogenesis in the adult rat: electron microscopic analysis of light radioautographs. Science.

[3] Gould E (1999). Hippocampal neurogenesis in adult Old World primates. Proc. Natl. Acad. Sci. USA.

[4] Eriksson PS (1998). Neurogenesis in the adult human hippocampus. Nat. Med..

[5] Winner B, Winkler J (2015). Adult neurogenesis in neurodegenerative diseases. Cold Spring Harb Perspect Biol.

[6] Sahay A, Hen R (2007). Adult hippocampal neurogenesis in depression. Nat. Neurosci..

[7] Jessberger S, Parent JM (2015). Epilepsy and Adult Neurogenesis. Cold Spring Harb Perspect Biol.

[8] Yu T-S, Washington PM, Kernie SG (2016). Injury-Induced Neurogenesis: Mechanisms and Relevance. Neuroscientist.

[9] Faigle R, Song H (1830). Signaling mechanisms regulating adult neural stem cells and neurogenesis. Biochim. Biophys. Acta.

[10] Gonçalves JT, Schafer ST, Gage FH (2016). Adult Neurogenesis in the Hippocampus: From Stem Cells to Behavior. Cell.

[11] Islam SM (2009). Draxin, a repulsive guidance protein for spinal cord and forebrain commissures. Science.

[12] Ahmed G (2010). Olfactory bulb axonal outgrowth is inhibited by draxin. Biochem. Biophys. Res. Commun..

[13] Hossain M (2013). The combinatorial guidance activities of draxin and Tsukushi are essential for forebrain commissure formation. Dev. Biol..

[14] Su Y (2009). Draxin, an axon guidance protein, affects chick trunk neural crest migration. Dev. Growth Differ..

[15] Su Y (2010). Draxin is involved in the proper development of the dI3 interneuron in chick spinal cord. Dev. Dyn..

[16] Naser IB (2009). Analysis of a repulsive axon guidance molecule, draxin, on ventrally directed axon projection in chick early embryonic midbrain. Dev. Biol..

[17] Riyadh MA, Shinmyo Y, Ohta K, Tanaka H (2014). Inhibitory effects of draxin on axonal outgrowth and migration of precerebellar neurons. Biochem. Biophys. Res. Commun..

[18] Shinmyo Y (2015). Draxin from neocortical neurons controls the guidance of thalamocortical projections into the neocortex. Nat Commun.

[19] Zhang S, Su Y, Gao J, Zhang C, Tanaka H (2017). A potential inhibitory function of draxin in regulating mouse trunk neural crest migration. In Vitro Cell. Dev. Biol. Anim..

[20] Zhang S (2010). Draxin, a repulsive axon guidance protein, is involved in hippocampal development. Neurosci. Res..

[21] Ahmed G (2011). Draxin inhibits axonal outgrowth through the netrin receptor DCC. J. Neurosci..

[22] Matsunaga E (2004). RGM and its receptor neogenin regulate neuronal survival. Nat. Cell Biol..

[23] Mehlen P, Mazelin L (2003). The dependence receptors DCC and UNC5H as a link between neuronal guidance and survival. Biol. Cell.

[24] Castets M (2012). DCC constrains tumour progression via its dependence receptor activity. Nature.

[25] Forcet C (2001). The dependence receptor DCC (deleted in colorectal cancer) defines an alternative mechanism for caspase activation. Proc. Natl. Acad. Sci. USA.

[26] Llambi F, Causeret F, Bloch-Gallego E, Mehlen P (2001). Netrin-1 acts as a survival factor via its receptors UNC5H and DCC. EMBO J..

[27] Villanueva AA (2017). The Netrin-4/Neogenin-1 axis promotes neuroblastoma cell survival and migration. Oncotarget.

[28] Hsieh J (2012). Orchestrating transcriptional control of adult neurogenesis. Genes Dev..

[29] Li G, Kataoka H, Coughlin SR, Pleasure SJ (2009). Identification of a transient subpial neurogenic zone in the developing dentate gyrus and its regulation by Cxcl12 and reelin signaling. Development.

[30] Naruse I, Keino H, Kawarada Y (1994). Antibody against single-stranded DNA detects both programmed cell death and drug-induced apoptosis. Histochemistry.

[31] Kawarada Y, Miura N, Sugiyama T (1998). Antibody against single-stranded DNA useful for detecting apoptotic cells recognizes hexadeoxynucleotides with various base sequences. Journal of Biochemistry.

[32] Watanabe I (2005). Detection of Apoptotic Cells in Human Colorectal Cancer by Two Different in situMethods: Antibody against Single-stranded DNA and Terminal Deoxynucleotidyl Transferase-mediated dUTP-biotin Nick End-labeling (TUNEL) Methods. Japanese Journal of Cancer Research.

[33] Maeda M (1998). Single stranded DNA as an immunocytochemical marker for apoptotic change of ischemia in the gerbil hippocampus. Neurosci. Lett..

[34] Mehlen P (1998). The DCC gene product induces apoptosis by a mechanism requiring receptor proteolysis. Nature.

[35] Furne C, Rama N, Corset V, Chédotal A, Mehlen P (2008). Netrin-1 is a survival factor during commissural neuron navigation. Proc. Natl. Acad. Sci. USA.

[36] Takemoto M (2011). Laminar and areal expression of unc5d and its role in cortical cell survival. Cereb. Cortex.

[37] Gao X (2015). A Floor-Plate Extracellular Protein-Protein Interaction Screen Identifies Draxin as a Secreted Netrin-1 Antagonist. Cell Rep.

[38] Haddick PCG (2014). Defining the ligand specificity of the deleted in colorectal cancer (DCC) receptor. PLoS ONE.

[39] Favaro R (2009). Hippocampal development and neural stem cell maintenance require Sox2-dependent regulation of Shh. Nat. Neurosci..

[40] Lavado, A., Lagutin, O. V., Chow, L. M. L., Baker, S. J. & Oliver, G. Prox1 is required for granule cell maturation and intermediate progenitor maintenance during brain neurogenesis. *PLoS Biol*. **8** (2010). 10.1371/journal.pbio.1000460 PMC2923090 20808958

[41] Roybon L (2009). Neurogenin2 directs granule neuroblast production and amplification while NeuroD1 specifies neuronal fate during hippocampal neurogenesis. PLoS ONE.

[42] Arnold SJ (2008). The T-box transcription factor Eomes/Tbr2 regulates neurogenesis in the cortical subventricular zone. Genes Dev..

[43] Kuwabara T (2009). Wnt-mediated activation of NeuroD1 and retro-elements during adult neurogenesis. Nat. Neurosci..

[44] Lie DC (2005). Wnt signalling regulates adult hippocampal neurogenesis. Nature.

[45] Qu Q (2013). Wnt7a regulates multiple steps of neurogenesis. Mol. Cell. Biol..

[46] Yoshinaga Y (2010). Wnt3a promotes hippocampal neurogenesis by shortening cell cycle duration of neural progenitor cells. Cell. Mol. Neurobiol..

[47] Miyake A (2012). Neucrin, a novel secreted antagonist of canonical Wnt signaling, plays roles in developing neural tissues in zebrafish. Mech. Dev..

[48] Miyake A (2009). Neucrin is a novel neural-specific secreted antagonist to canonical Wnt signaling. Biochem. Biophys. Res. Commun..

[49] Little GE (2009). Specificity and plasticity of thalamocortical connections in Sema6A mutant mice. PLoS Biol..

