# Supplementary material for: Draxin regulates hippocampal neurogenesis in the postnatal dentate gyrus by inhibiting DCC-induced apoptosis
Source: Sci Rep. 2018 Jan 16;8:840. doi: 10.1038/s41598-018-19346-6 (PMC5770412; doi:10.1038/s41598-018-19346-6)
Supplement: Supplementary file 1 — Supplementary information [file 41598_2018_19346_MOESM1_ESM.pdf]

Draxin regulates hippocampal neurogenesis in the postnatal dentate gyrus by inhibiting DCC-induced apoptosis

Hiroshi Tawarayama[1,2,3], Hirohisa Yamada[1], Ruhul Amin[2], Yuiko Morita-Fujimura[2,4], Helen M Cooper[5], Yohei Shinmyo[1], Masakado Kawata[3], Shuntaro Ikawa[2], Hideaki Tanaka[1]

[1] Department of Developmental Neurobiology, Graduate School of Medical Sciences, Kumamoto University, Kumamoto 860-8556, Japan

[2] Department of Project Programs, Institute of Development, Aging and Cancer (IDAC), Tohoku University, Sendai 980-8575, Japan

[3] Department of Ecology and Evolutionary Biology, Graduate School of Life Sciences, Tohoku University, Sendai 980-8578, Japan

[4] Frontier Research Institute for Interdisciplinary Sciences (FRIS), Tohoku University, Sendai 980-8578, Japan

[5] The University of Queensland, Queensland Brain Institute, Brisbane, Queensland 4072, Australia

Corresponding author: Hiroshi Tawarayama; Department of Project Programs, Institute of Development, Aging and Cancer (IDAC), Tohoku University, 4-1 Seiryo-machi, Aoba-ku, Sendai 980-8575, Japan

Phone: +81-22-717-8471; Fax: +81-22-717-8476

E-mail: hiroshi.tawarayama.b4@tohoku.ac.jp

EXPERIMENTAL PROCEDURES

Measuring the area size of the dentate gyrus. Brains, fixed with 4 % paraformaldehyde (PFA; Wako Pure Chemical) in PBS, were dissected from various developmental stages of mice, and then cut into 18 μm-thick coronal sections using a cryostat (Leica). To compare the area size of the dentate gyrus (DG) at the similar histological location between *draxin* wild-type (WT) and knockout (KO) mice, sections containing the habenular nucleus were collected, and then immersed in Mayer's hematoxylin (Sigma-Aldrich) and 1 % eosin solutions (Wako Pure Chemical) to visualize the DG structure. After taking photos of the DG, its area size was measured using ImageJ software provided by the National Institutes of Health (NIH), and shown as a percentage of the area size in the WT DG.

Immunochemical analyses. Immunohistochemistry on hippocampal sections was performed as described previously (Tawarayama et al., 2010). Briefly, dissected whole brains were fixed with 4 % paraformaldehyde (PFA; Wako Pure Chemical) in PBS overnight at 4 °C, and then cut into 18- or 80 μm-thick sections using a cryostat (Leica) or vibratome (Leica), respectively. Sections were treated with 5 % normal donkey serum

(Merck-Millipore) in PBS containing 0.1 % Triton-X100 to block non-specific binding, then reacted with primary antibodies overnight at 4 °C. After removing the primary antibodies, sections were incubated with fluorophore- or horseradish peroxidase (HRP)-conjugated secondary antibodies for 1 hour at room temperature. Color development was done using the Vector VIP Peroxidase Substrate Kit (Vector laboratories). Immunocytochemistry was performed following the immunohistochemical procedure described above with a modified fixation time (30 min). The details for antibodies and fluorophores used in immunochemical analyses are shown in the Supplementary Table.

Expression analysis of neogenin in the postnatal subgranular zone (SGZ). To examine neogenin expression in the DG, we used a mutant mouse line, in which a ß-gal gene-trapping vector was integrated into one of the neogenin alleles (Leighton et al., 2001; Mitchell et al., 2001). In this mutant line, truncated neogenin-ß-gal fusion proteins are generated and trapped in the endoplasmic reticulum of the expressing cells, where they appear as punctate and perinuclear ß-gal immunoreactivity. Thus, the cells immunoreactive to ß-gal were regarded as neogenin-expressing cells. To determine the cell type of SGZ cells expressing neogenin, hippocampal slices from P30 neogenin

heterozygous mice were coimmunostained for ß-gal and expression markers, specific to various differentiation stages in the granule cell lineage, including GFAP, nestin, Tbr2, NeuroD1 and Prox1.

Evaluation of efficiency of siRNA transfection and protein knockdown. To evaluate transfection efficiency of siRNA duplexes, the siGLO-Red transfection indicator (GE Healthcare) was introduced into rat HNSPCs using Lipofectamine RNAiMAX Reagent (Invitrogen) at a final concentration of 20 nM. HNSPCs were fixed with 4 % PFA in PBS one day after transfection, followed by counter staining with DAPI. Number of cells incorporating siGLO-Red was counted and shown as a percentage of that of total cells. To evaluate protein knockdown efficiency of siRNA duplexes specific to DCC and neogenin, these siRNA duplexes (20 nM) were introduced into HNSPCs, pretreated with differentiation inducers for one day to induce expression of DCC and neogenin, using Lipofectamine RNAiMAX Reagent. Next day, whole cell lysates were extracted from the transfectants, and then expression level of each target protein was analyzed by the Western blot analysis.

Western blot analyses. To perform the Western blot analysis, rat hippocampal neural

stem/progenitor cells (HNSPCs) were cultured in 6-well plates in various conditions, and lysed in RIPA buffer containing protease inhibitors (Cayman chemical). Lysate protein concentrations were determined by the BCA Protein Assay Kit (Pierce), and then 15 μg of protein was used for the western blot analysis. Electrophoresis and membrane blotting were performed according to routine protocols. The details for antibodies used in the Western blot analysis are shown in the Supplementary Table.

Figure Legends

Supplementary Figure S1. Impaired development of the DG in *draxin*-deficient mice. ***A***, Hematoxylin and eosin staining of hippocampal sections of *draxin* wild-type (WT) and knockout (KO) mice at various developmental stages: Embryonic day (E) 17.5, postnatal day (P) 0, P15, and adult. The region of the DG was delineated by while lines. ***B***, The area of the DG shown as a percentage relative to WT. The average was calculated from 5 independent brains per group. Error bars indicate s.e.m.. $^{*}p < 0.05$, $^{**}p < 0.01$ against wild-type mice (Student's t-test). CA: Cornu Ammonis; DG: dentate gyrus; gcl: granule cell layer; h: hilus; pcl: pyramidal cell layer. Scale bar, 200 μm.

Supplementary Figure S2. Expression of neogenin in the postnatal SGZ. ***A-E,*** Double immunohistochemistry on the DG of P30 for ß-gal, which is expressed as a fused protein with neogenin (see the supplementary text above for the detail), and various markers of the granule cell lineage including GFAP (***A***,***A'***,***A''***), nestin (***B***,***B'***,***B''***), Tbr2 (***C***,***C'***,***C''***), NeuroD1 (***D***,***D'***,***D''***) and Prox1 (***E***,***E'***,***E''***). Confocal images of each marker and ß-gal, and their merges were shown in ***A-E***, ***A'-E'*** and ***A''-E''***, respectively. ***F,*** Quantification of ß-gal (+) cells expressing each of various markers. Arrowheads indicate cells doubly expressing ß-gal and each marker. gcl: granule cell layer; h: hilus. Scale bar, 20 μm.

Supplementary Figure S3. Expression profile of DCC and neogenin in rat hippocampal neural stem and progenitor cells (HNSPCs) during differentiation. ***A,*** Immunostaining of HNSPCs for nestin and Tuj1. Rat HNSPCs were treated with retinoic acid (RA) and forskolin (FK) to stimulate neuronal differentiation for four days, and then expression of nestin and Tuj1, markers for progenitor and young neurons, respectively, was examined by immunocytochemistry. Note that almost all the rat HNSPCs express Tuj1 on the 4th day of treatment with differentiation inducers, indicating that fate of the cells is committed to neurons in the presence of RA/FK. ***B,*** The western blot analysis of DCC,

neogenin and ß-actin on cell lysates prepared from differentiating HNSPCs. Protein-transferred PVDF membrane were separated into two parts, the upper and lower, and then DCC (left) or neogenin (right) and ß-actin (both) were detected using antibodies, respectively. Scale bar, 50 μm.

Supplementary Figure S4. Efficiency of siRNA transfection and protein knockdown. ***A***, Rat HNSPCs transfected with the siGLO-Red transfection indicator. ***B***, Quantification of transfected cells in ***A***. ***C***, Western blot analysis of DCC and neogenin on HNSPCs transfected with siRNA duplexes specific to DCC (si-DCC), neogenin (si-neogenin), or control siRNA duplexes (si-control). Cell lysates prepared from siRNA duplex transfectants were transferred on PVDF membranes. The membranes were separated into the upper and lower parts, and then expression of DCC (left) or neogenin (right) and ß-actin (both) were examined using antibodies, respectively. Experiments were performed in triplicate. ***D***, Quantification of immunoblot signals in ***C***. Expression level of DCC or neogenin in siRNA-transfectants was shown as a percentage against that in si-control-transfectants. The average was calculated from 3 independent experiments. Error bars indicate s.e.m.. *$p < 0.01$ (Student's t-test). Scale bar, 50 μm.

Supplementary Table. Information of the antibodies and fluorophore used in this study.

Primary antibodies				
Antigens	Supplier	Catalog #	Host	Dilution
draxin	R&D Systems	AF6149	sheep	100
ß-galactosidase	Abcam	ab9361	chicken	500
GFAP	DAKO	IR524	rabbit	2,000
nestin	EDM Millipore	MAB353	mouse	500
Sox2	Santa Cruz Biotechnology	sc-17320	goat	100
Tbr2	Abcam	ab23345	rabbit	500
NeuroD1	Santa Cruz Biotechnology	sc-1084	goat	100
DCX	Santa Cruz Biotechnology	sc-8066	goat	100
Prox1	EDM Millipore	AB5475	rabbit	1,000
NeuN	EDM Millipore	MAB377	mouse	500
S100ß	Sigma-Alldrich	S2532	rabbit	500
single-stranded DNA	Immuno-Biological Laboratories	18731	rabbit	100
active caspase-3	Cell Signaling Technology	9664	rabbit	1,000
HA	Sigma-Aldrich	H9658	mouse	1,000
FLAG	Sigma-Aldrich	F3165	mouse	1,000
GFP	Nacalai Tesque	4363-24	rat	1,000
Tuj1	R&D Systems	MAB1195	mouse	1,000
GAPDH	Gene Tex	GTX100118	rabbit	2,000
active ß-catenin	Cell Signaling Technology	8814	rabbit	5,000
Ki-67	Spring Bioscience	M3060	rabbit	500
neogenin (C-20)	Santa Cruz Biotechnology	sc-6536	goat	100
DCC (A-20)	Santa Cruz Biotechnology	sc-6535	goat	100
ß-actin (AC-74)	Sigma-Aldrich	A5316	mouse	2,000

Secondary antibodies				
Name	Supplier	Catalog #	Host	Dilution
AF488 anti-mouse IgG	Invitrogen	A-21202	donkey	500
AF488 anti-rat IgG	Invitrogen	A-21208	donkey	500
AF488 anti-rabbit IgG	Invitrogen	A-21206	donkey	500
AF488 anti-goat IgG	Invitrogen	A-11055	donkey	500
AF568 anti-goat IgG	Invitrogen	A-11057	donkey	500
Cy3 anti-mouse IgG	Jackson ImmunoReseearch	715-165-150	donkey	500
Cy3 anti-rabbit IgG	Jackson ImmunoReseearch	711-165-152	donkey	500
Cy3 anti-goat IgG	Jackson ImmunoReseearch	805-165-180	donkey	500
Cy3 anti-chicken IgY	Jackson ImmunoReseearch	703-165-155	donkey	500
HRP anti-sheep IgG	Jackson ImmunoReseearch	713-035-147	donkey	1,000
HRP anti-goat IgG	R&D Systems	HAF017	donkey	10,000
HRP anti-mouse IgG	Jackson ImmunoReseearch	715-035-150	donkey	10,000
HRP anti-rabbit IgG	Jackson ImmunoReseearch	711-035-152	donkey	10,000
biotin anti-sheep IgG	Sigma-Aldrich	B7390	donkey	1,000
biotin anti-mouse IgG	Jackson ImmunoReseearch	715-065-150	donkey	1,000
biotin anti-rabbit IgG	Jackson ImmunoReseearch	711-065-152	donkey	1,000

Fluorosphere-conjugated streptavidin				
Name	Supplier	Catalog #	Host	Dilution
AF647 streptavidin	Invitrogen	S-213	-	1,000

References

Leighton, P. A., Mitchell, K. J., Goodrich, L. V., Lu, X., Pinson, K., Scherz, P., Skarnes, W. C. and Tessier-Lavigne, M. (2001). Defining brain wiring patterns and mechanisms through gene trapping in mice. *Nature* **410**, 174–179.

Mitchell, K. J., Pinson, K. I., Kelly, O. G., Brennan, J., Zupicich, J., Scherz, P., Leighton, P. A., Goodrich, L. V., Lu, X., Avery, B. J., et al. (2001). Functional analysis of secreted and transmembrane proteins critical to mouse development. **28**, 241–249.

Tawarayama, H., Yoshida, Y., Suto, F., Mitchell, K. J. and Fujisawa, H. (2010). Roles of semaphorin-6B and plexin-A2 in lamina-restricted projection of hippocampal mossy fibers. *J. Neurosci.* **30**, 7049–7060.

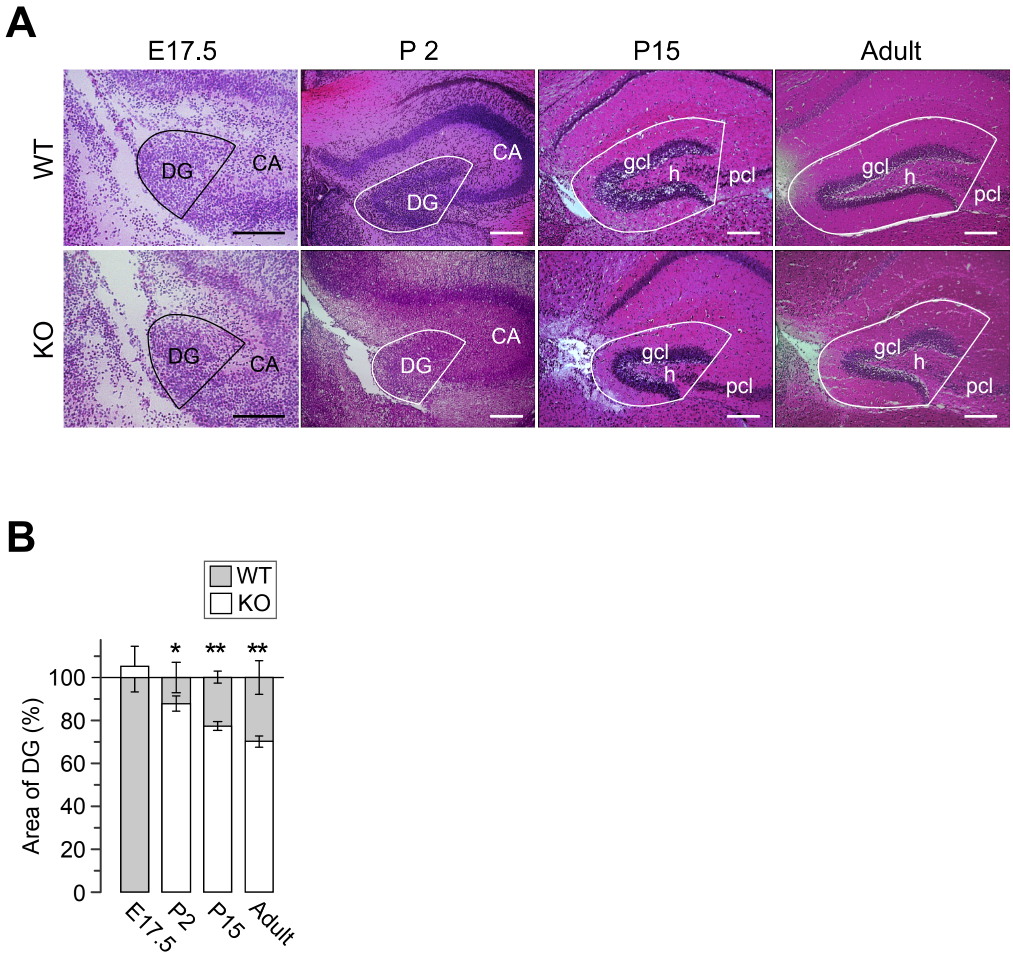

Supplementary Figure S1. Tawarayama *et al.*

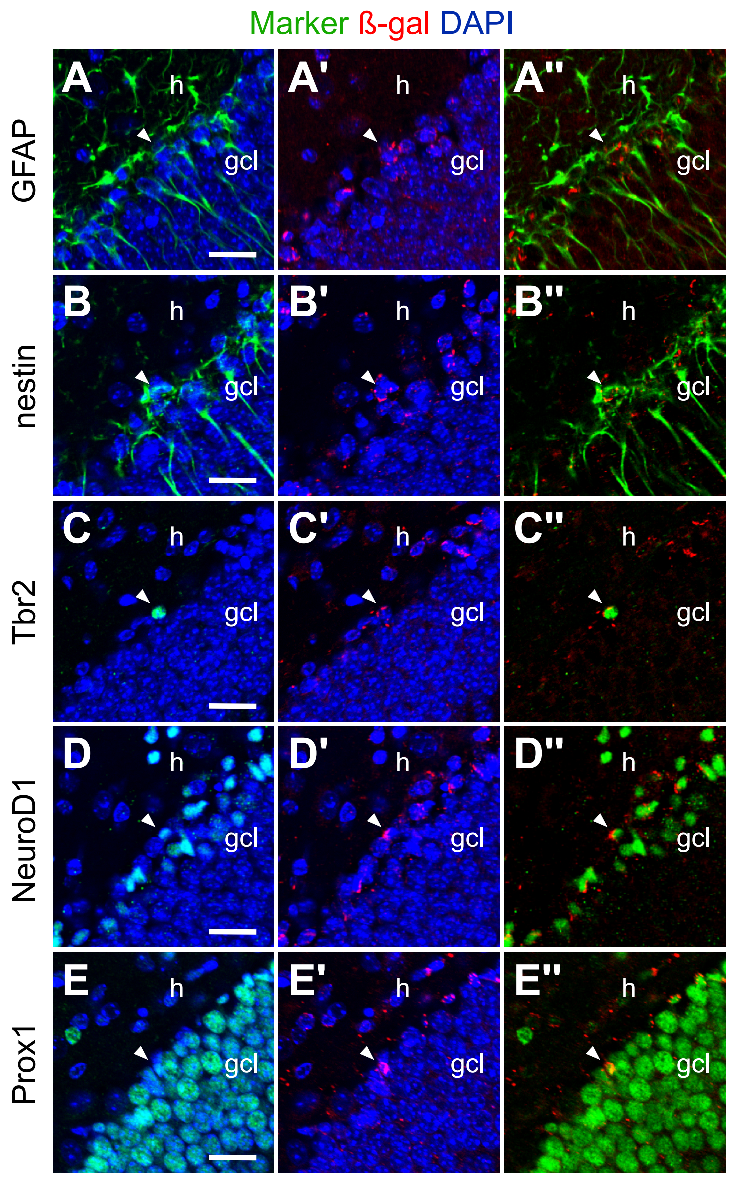

F

Marker (+) cells expressing ß-gal (%)	
GFAP	5.1
nestin	62.6
Tbr2	30.1
NeuroD1	32.0
Prox1	6.9

Supplementary Figure S2. Tawarayama *et al.*

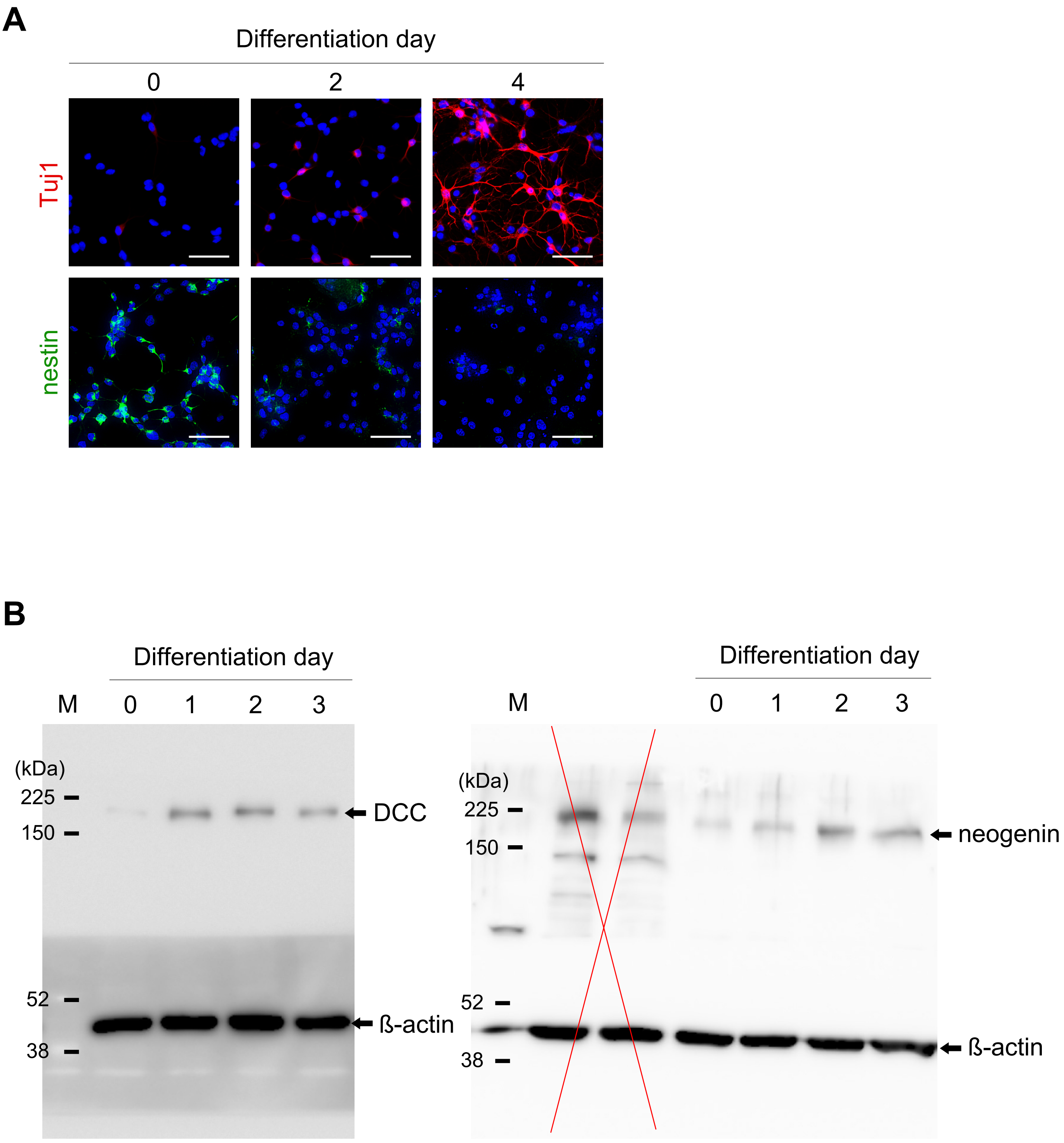

Supplementary Figure S3. Tawarayama *et al.*

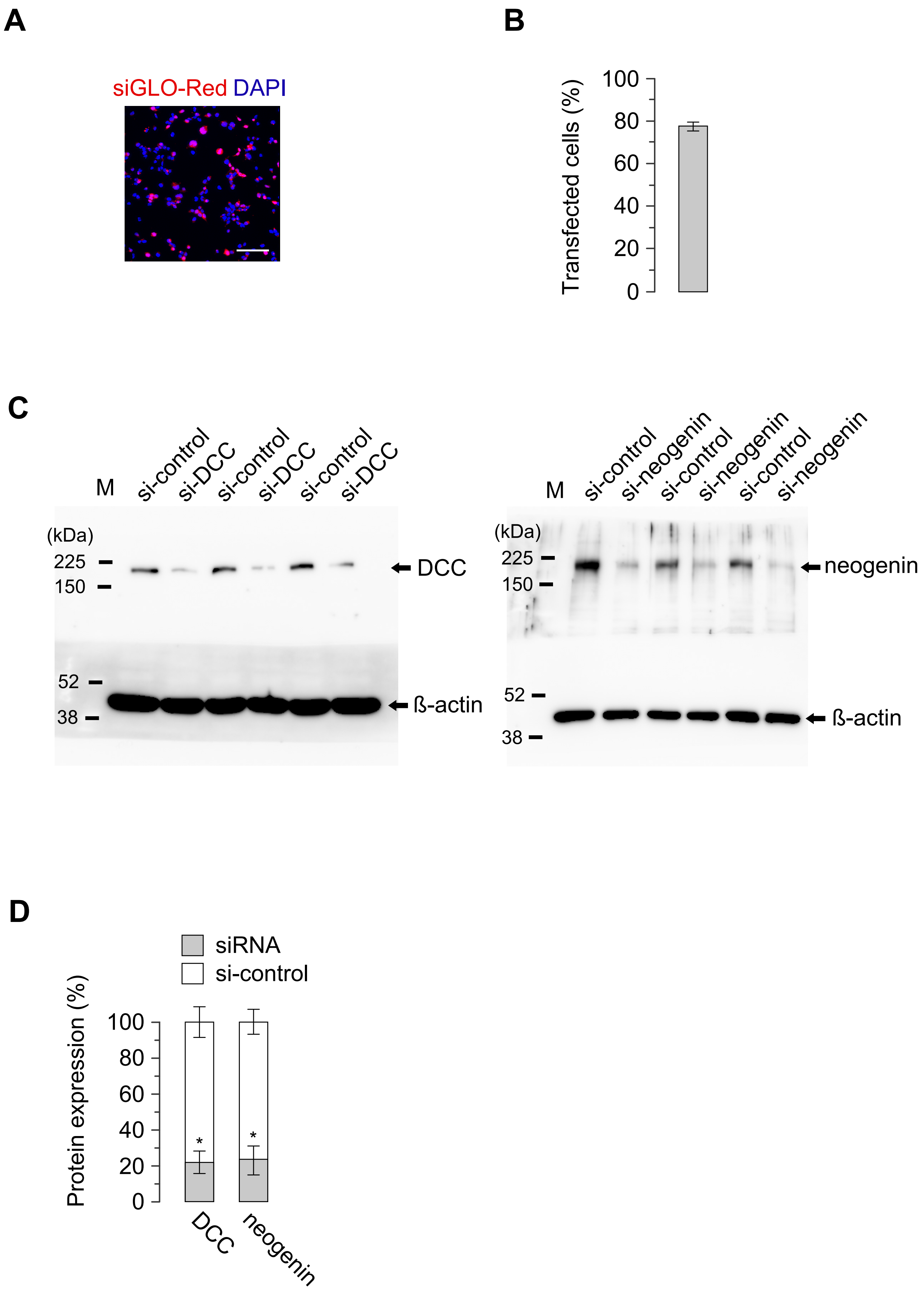

Supplementary Figure S4. Tawarayama *et al.*
